# Sex Differences Independent of Other Psycho-sociodemographic Factors as a Predictor of Body Mass Index in Black South African Adults

**DOI:** 10.3329/jhpn.v30i1.11277

**Published:** 2012-03

**Authors:** Annamarie Kruger, Maria P. Wissing, Gordon W. Towers, Colleen M. Doak

**Affiliations:** ^1^Africa Unit for Transdisciplinary Health Research and Faculty of Health Sciences, North-West University (Potchefstroom Campus), Potchefstroom, South Africa; ^2^Centre of Excellence for Nutrition, Faculty of Health Sciences, North-West University (Potchefstroom Campus), Potchefstroom, South Africa; ^3^Department of Health Sciences, Vrije Universiteit, Amsterdam, The Netherlands

**Keywords:** Body mass index, Body-weight, Cross-sectional studies, Gender, Overweight, Obesity, Sex difference, South Africa

## Abstract

To better understand the sex differences in body mass index (BMI) observed in black South African adults in the Transition and Health during Urbanization of South Africans Study, the present study investigated whether these differences can be explained by the psycho-sociodemographic factors and/or health-related behaviours. A cross-sectional survey was undertaken among 1,842 black South African individuals from 37 study sites that represented five levels of urbanization. The behavioural factors that possibly could have an influence on the outcome of body-weight and that were explored included: diet, smoking, level of education, HIV infection, employment status, level of urbanization, intake of alcohol, physical activity, and neuroticism. The biological factors explored were age and sex. The prevalence of underweight, normal weight, and overweight among men and women was separately determined. The means of the variables were compared by performing Student's *t*-test for normally-distributed variables and Mann-Whitney U-test for non-normally-distributed variables. The means for the underweight and overweight groups were tested for significant differences upon comparison with normal-weight individuals stratified separately for sex. The differences in prevalence were tested using chi-square tests (p<0.05). All the variables with a large number of missing values were tested for potential bias. The association between sex and underweight or overweight was tested using the Mantel-Haenszel method of odds ratio (OR) and calculation of 95% confidence interval (CI), with statistical significance set at p<0.05 level. Logistic regression was used for controlling for confounders and for testing for effect modification. Females were more likely to be overweight/obese (crude OR=5.1; CI 3.8-6.8). The association was attenuated but remained strong and significant even after controlling for the psycho-sociodemographic confounders. In this survey, the risk for overweight/obesity was strongly related to sex and not to the psycho-sociodemographic external factors investigated. It is, thus, important to understand the molecular roots of sex and gender-specific variability in distribution of BMI as this is central to the future development of treatment and prevention programmes against overweight/obesity.

## INTRODUCTION

The nutrition transition describes a pattern of dietary and activity changes often observed in countries experiencing economic growth (1,2). This pattern has been associated with the rising prevalence of overweight and obesity among urban populations, often co-existing with undernutrition (3-5). In South Africa, this clustering of undernutrition and overweight/obesity was first reported by Steyn *et al.* who determined a high prevalence of stunted children and overweight/obese women in the same low-income community (6). Later studies reported similar findings in countries experiencing rapid economic transition (7-9).

Studies in which the roles of various gender/sex-specific risk factors were explored in relation to the status of body-weight are rare, although a number of surveys in different countries indicate that there are gender/sex differences in weight status. The association of overweight and obesity specifically with women in the African context needs to be explained. Currently, it is not clear whether sex or gender differences, or both, play a role. To understand the sex/gender differences, proper clarification of these terms is needed. As used in recent literature, the concept ‘sex differences’ refers to those variables that are exclusively biological whereas the ‘gender differences’ refer to the socially-defined differences between men and women and by definition also include variables relating to possible interactions between the biological and the environmental factors (10). As this study will focus on the possible role played by the psycho-sociodemographic and behavioural variables (which may include interactions among biological and environmental variables), the concept of ‘gender differences’ is preferred in the discussion rather than ‘sex differences’.

Results of a study in South Africa on black teenagers showed that girls were particularly more prone to overweight/obesity compared to boys (11). Similar results from Africa, the Middle East (12-15), and Mexico (16) have been reported. However, surveys in Brazil showed that the prevalence of overweight/obesity in this country was similar for men and women. Results of studies, conducted during the 1990s, showed that, in China (17) and Malaysia (18), men, rather than women, had a greater prevalence of overweight/obesity. However, Kelly *et al.* showed a clear global pattern with women having a higher prevalence of overweight/obesity compared to men (19). Furthermore, Mendez *et al.* showed that overweight in females already exceeded underweight in most developing countries (20).

The sex differences in metabolic rate are well-documented, and studies have demonstrated the gender-specific differences in dietary intake, smoking, and physical activity (21). However, no studies have explored the role of behaviour in explaining differing risks for men and women relating to underweight and overweight. The Transition and Health during Urbanization of South Africans (THUSA) Study, which focuses on a community experiencing the nutrition transition, provides an ideal population for exploring this question. Kruger *et al.* reported a high prevalence of overweight/obesity among women in the THUSA study (22). Furthermore, several psycho-sociodemographic and behavioural variables, including facets, such as neuroticism, were included and found to be significantly different over levels of urbanization (23). Data from 37 nations indicated that women had higher levels of neuroticism (24). Costa *et al*. confirmed this finding in data from 26 cultures but they also noted that, contrary to the predictions from an evolutionary perspective, the magnitude of the gender differences varied across cultures and that, contrary to a social role perspective, the gender differences were surprisingly greater in European and American samples in which the traditional gender roles are minimized (25). Faith *et al*. determined in a British sample that an increase in body mass index (BMI) was significantly associated with neuroticism in women only (26). In a large prospective study on the association of psychosocial factors with health and disease, Brummett *et al*. confirmed that neuroticism was related to BMI in women only (27). They argued that neuroticism can be an endogenous causal factor of obesity but Chrisler and McCreary pointed out that neuroticism may also be a reaction to the social context with its pressure on women to be slim within which the neuroticism is expressed (28). In an African context, obesity has traditionally been viewed as a sign of prosperity and health, although it is currently more often a sign of a poverty-related poor diet (29,30). It is still unknown what the interactions among gender, neuroticism, and BMI in an adult black African group will be.

Therefore, understanding the gender and sex-specific underweight and overweight determinants and their interactions is a critical first step towards potentially resolving the dual burden of undernutrition and overweight/obesity.

## MATERIALS AND METHODS

### Study subjects

The THUSA study was a cross-sectional survey of 1,842 individuals from 37 study sites that represented five levels of urbanization. The original sample design, selection of participants, and ethical practices were previously published (23). Fifty subjects were included from each of the following age-groups: 15-<25, 25-<35, 35-<45, 45-<55, 55-<65, and ≥65 years.

Due to the complexities of comparing results based on BMI classifications of the older and younger groups, the youngest group (15-<25 years) (16-23, 31) and the eldest group (≥65) were excluded. The remaining four age-groups comprised the study subjects, with 1,325 individuals aged 25-64 years.

### Procedure

The results found from male and female adults were compared in a descriptive manner to determine if the statistically significant determinants for obesity in females were the same in males and vice-versa.

The biological determinants investigated were age and sex while the behavioural determinants were diet, dependants sharing food, employment status, smoking, education, HIV infection, intake of alcohol, level of neuroticism, and physical activity. All measurements in the THUSA study were done using the appropriate validation and quality-control procedures and have been previously published (23).

### Exposure

**Weight status:** Anthropometric measurements were taken by postgraduate students under the supervision of a level III anthropometrist. The weight status in this investigation was measured according to the BMI categories of the World Health Organization (WHO) for underweight, normal weight, and overweight/obesity. The WHO cut-off values for adults are defined as a BMI of <18.5 kg/m^2^ for underweight,18.5-<25 kg/m^2^ for normal weight, and 25 kg/m^2^ and above for classifying overweight and obesity together.

### Determinants investigated

**Diet:** The differences in the mean intake of total fibre, intake of total fruit and vegetable, energy, and percentage of energy from protein, carbohydrate, and fat between men and women were determined using the dietary intakes (including alcohol) as collected from a culturally-sensitive quantified food-frequency questionnaire (QFFQ), which was developed and validated by MacIntyre *et al*. (32,33). The respondents estimated portion-sizes using a food portion photograph book developed and tested for use in the African population of the North West province of South Africa (34). Portion-sizes were reported in household measures and were converted to weights using standard tables. The food intake was coded using the new food codes of the South African food-composition database of the South African Medical Research Council and was expressed as average amounts consumed per day (23).

**Smoking:** Smoking was expected to be associated with body-weight (35). Therefore, the gender differences in smoking were compared. The respondents were asked whether they currently smoke. The gender differences were compared according to category of smoking and using logistic models. Possible confounding due to smoking status was tested in association with underweight or overweight.

**Education:** Level of education was also a possible factor associated with body-weight status and was compared between men and women. Three levels of educational status were determined as follows: (a) no education; (b) lower than 10th grade; and (c) 10th grade and above.

**HIV status:** HIV is associated with underweight, and therefore, HIV infection may differ between men and women. The status of HIV was, thus, tested as a possible confounder. The status of HIV was determined using a ‘rapid test’ enzyme-immunological method (Enzymun Test®, anti-HIV 1+2+subtypeΦ, Boehringer Mannheim, Mannheim, Germany) (23).

**Age:** The study subjects within the age-categories of 15-<25, 25-<35, 35-<45, 45-<55, 55-<65, and ≥65 years were included. As the study design for THUSA already accounts for age by stratum, comparisons between strata were adjusted for age. Given the sampling design, age was not controlled for in the analysis as a confounder. However, the possible interactions between age and gender were explored.

**Employment:** The study subjects were asked to identify themselves as having a job or not. This was used as a dichotomous variable called ‘job at moment’. The possible confounding interactions were determined by exploring whether results from the level of urbanization differed between men and women for those who were unemployed compared to those who had a job at the time of the study.

**Level of urbanization:** The level of urbanization was indicated according to the following five strata: (i) those living in commercial farms, (ii) under tribal law (rural), (iii) in an urban informal housing sector, (iv) in an urban established housing area, or (v) in an upper income urban neighbourhood. This analysis compared the prevalence of underweight and overweight individuals by sex for each level of urbanization. A linear relationship between the body-weight outcomes and the urbanization levels was not expected because of the socioeconomic differences within each of these environments. Therefore, each stratum was analyzed separately, and the reference group chosen was based on the most extreme differences.

**Alcohol intake:** Consumption of alcohol was categorized as high, moderate, and low based on the upper and lower limits of the current guidelines for the intake of moderate alcohol (36). This information was used for creating a per-diem recommendation. An intake of ≥30 g of alcohol by men was, thus, used as a cut-off for the high intake group compared to those who consumed less than 30 g. The cut-off limit for women was 15 g for the high intake group compared to those who consumed <15 g. A high intake of alcohol, i.e. above the level recommended as moderate, was used for determining any associations between the intake of alcohol and underweight or overweight.

**Physical activity:** Levels of physical activity were based on the physical activity index developed and validated for use in this specific population (37). An index with a value from 1 to 3.3 was taken as low physical activity, an index with a value from 3.34 to 6.67 as moderate physical activity, and an index with a value higher than 6.67 as high physical activity (22). The cut-off value used for physical activi-ty in this investigation was set at 2.5. This value was equivalent to the median for men and the 75th percentile for women. This allowed the maximization of comparability between men and women while still having sufficient samples in the analyses. Men and women with activity levels of above 2.5 were categorized as having high activity.

**Having a child younger than 11 years at home:** This variable was used for giving an indication of the presence of dependants who share a meal without any contribution. This influenced the portion-sizes of the remaining family members, especially the caregivers of those children.

**Neuroticism:** Neuroticism was measured by the Setswana-validated neuroticism subscale (N) of the revised NEO personality inventory (NEO-PI-R) of Costa and McCrae (38). N is an index of negative effects as manifested in fear, sadness, embarrassment, anger, guilt, and disgust. People with high scores on N cope more poorly than others with stress. A Cronbach's alpha reliability index of 0.86 was obtained. To test for stress as an explanation of the associations determined, all odds ratios (ORs) were tested for confounding using the highest tertile of the neuroticism index.

### Statistical methods

The first analysis undertaken was to determine the prevalence of underweight, normal weight, and overweight among men and women separately. Simultaneously, the association between gender and underweight or overweight was tested using the Mantel-Haenszel method of OR and calculation of 95% CI, with statistical significance set at the p<0.05 level. Results of preliminary analysis indicated that all the variables, except the percentage of energy intake from fat, protein, and carbohydrate, were not normally distributed. The mean comparisons of the non-normally-distributed variables were obtained using the Mann-Whitney test. A preliminary exploration of all the variables was undertaken based on the mean differences for the continuous variables. The means for underweight and overweight groups were tested for significant differences upon comparison with normal-weight individuals. For dichotomous comparisons, the chi-square test was used. All the initial statistical comparisons were used for testing the differences among the overweight, underweight and normal-weight groups stratified separately by sex. The differences in prevalence were tested using the chisquare test (p<0.05).

All the variables with a large number of missing values were explored for potential bias. Bias was assessed by comparing the results for the study subjects with and without the values missing for a given variable.

Sets of questionnaire to determine the level of physical activity and neuroticism were completed for only a sub-sample of the participants in the THUSA study. It was determined that the sample was biased due to the fact that these variables were collected mostly in urban areas. To avoid introducing the possible bias by controlling for a variable that was collected on a sub-sample that differed from the total population, the models used in this investigation were not controlled for physical activity and neuroticism.

Based on a 10% change in the OR, there was no confounding effect determined for the intake of fibre, consumption of alcohol, education, status of HIV, age-group, having a child younger than 11 years at home, and the urbanization level of an individual. The smoking status did not confound the association between female sex and overweight/obesity but did partially explain the association between gender and underweight. The protective association between female sex and underweight was attenuated and was no longer significant after controlling for the smoking status (OR=0.67; CI 0.44-1.02). For this reason, smoking was controlled for in further analyses.

### Ethical approval

The Ethics Committee of the former Potchefstroom University for Christian Higher Education approved the study, and all participants gave written informed consent.

## RESULTS

**Fig. 1.**
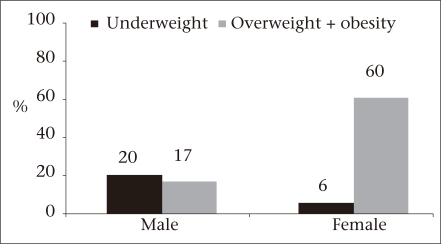
Prevalence of underweight and overweight ⩲ obesity by gender

Figure 1 presents the stacked prevalence of underweight, normal weight, overweight, and obesity for men and women included in the study. These results indicate a higher prevalence of underweight among males (20.2%) and overweight/obesity among females (60.3%).

**Fig. 2.**
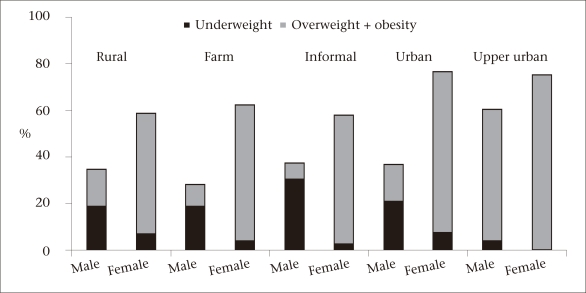
Prevalence of weight status by gender and stratum of urbanization

Figure 2 depicts a clear and consistent sex pattern in which men had a high prevalence of underweight in all the strata of urbanization, except the upper-income urban areas whereas women had a higher prevalence of overweight in all the strata of urbanization. In the informal sector, 30.7% of the men were underweight while 55.1% of the women were either overweight or obese. In the total sample, only 22 males were categorized as obese.

The measured baseline characteristics that might influence the prevalence of obesity for men and women are presented in Table 1 and 2. The results indicate that women had a higher mean BMI [27.8 kg/m^2^ ±standard deviation (SD) of 7], a higher percentage of energy from the intake of fat (25.6% SD±7.3), and a higher mean neuroticism index (171.1 SD±21.4) than men (21.5 kg/m^2^ SD±4.1; 24.5% SD±7.6; and 167.3 SD±21.6 respectively). Men, however, had a significantly higher mean intake of fibre (17.4 g/day SD±8.9), total energy (9,377.5 Kj SD±3,884.8), a percentage of energy from alcohol (5.6 g/day SD±8.3), and a higher mean physical activity score (3.4 SD±1.6) than women (16 g/day SD±7.4; 7,818.7 Kj SD±3,058.5; 1.2 g/day SD±3.7; and 2.6 SD±0.7 respectively) (Table 1).

The prevalence of current smokers was three times higher among men (65.5% vs 20.1%) than among women. Furthermore, more men had no education (p=0.003) while women were more likely to have a middle level of education (p=0.012). No significant differences in the frequency of higher education (p=0.902) were determined between men and women. Thirteen percent of the men and 11% of the women were identified as being infected with HIV in this population. Consistent with the study design, there were no differences between men and women when categorized according to age. Proportionately, more men were employed at the time of the study compared to women (60.7% vs 42.1%, p<0.001) whereas more men had a high level of alcohol intake (22.8% vs 6.9%, p<0.001) and a higher activity level (66.4% vs 41.6%, p<0.001). Although only half of the study sample had values for the neuroticism index, the gender differences were significant. Proportionally, more females had scores for neurosis in the third and fourth quartiles (p=0.008) (Table 2).

The results for the stratum-specific ORs for females after controlling for smoking status are presented in Table 3. In all the strata, being female was strongly and significantly associated with overweight/obesity. However, the ORs were not same within all the groups. The association between female sex and overweight/obesity was stronger if there was “no child younger than 11 years at home” compared to when there was a child aged 11 years in the home. In all the age-groups (except for the 45-<55 years), obesity was 5.9 to 7.3 times stronger among females. Given the differences in the strength of the association across the levels of strata, ‘hav ng a child younger than 11 years at home’ and ‘age 45-<55 years’ were selected as the possible effect modifiers.

The second column of Table 3 presents the association between gender/sex and underweight, indicating a consistent protective association between female sex and underweight. However, the only statistically significant finding was the association between female sex and underweight (OR=0.44) in rural areas, farms, and the informal sector (CI 0.26-0.75). Alternatively, in the urban residents (including those residing in upper-income areas), female sex was positively associated (OR=1.97; CI 0.92-4.22) with underweight, albeit non-significantly. Again, when the stratum-specific ORs were further controlled for physical activity and neuroticism, these results were amplified rather than diminished (data not indicated). Given these very different effects of sex in relation to underweight, ‘urban residence’ was selected as a possible effect modifier.

**Table 1.** Baseline characteristics of men and women: means (SD)

Variable	Men (n=530) Mean (SD)	Women (n=795) Mean (SD)
BMI* (kg/m^2^)	21.5 (4.1)	27.8 (7.0)
Fibre* (g/day)	17.4 (8.9)	16.0 (7.4)
Fruit and vegetable (g/day)	2.1 (1.8)	2.4 (2.2)
Total energy*(kJ)	9,377.5 (3,884.8)	7,818.7 (3,058.5)
% of food energy from protein	12.1 (2.1)	11.9 (2.3)
% of food energy from fat**	24.5 (7.6)	25.6 (7.3)
% of food energy from carbohydrate	65.1 (9.9)	64.1 (9.9)
% of energy from alcohol**	5.6 (8.3)	1.2 (3.7)
Physical activity index* (Men=281 and women=387)	3.4 (1.6)	2.6 (0.7)
Neuroticism index* men n=246 women (n=328)	167.3 (21.6)	171.1 (21.4)

*p<0.001;

**p<0.05;

BMI=Body mass index;

SD=Standard deviation

Table 4 explores the effect modification for variables selected based on the above analysis, thus ‘having a child younger than 11 years at home’, ‘age of 45-<55 years’, and ‘urban residence’ were tested as the effect modifiers of the relationship of sex with BMI. The results were tested using a logistic model while controlling for the confounding effect of smoking status (current smoker). Using the criteria of α<0.10 for the interaction term, the results indicate that female sex was associated with an increased risk of overweight/obesity in all the strata (p=0.05). The interaction term shows that ‘having a child younger than 11 years at home’ was an effect modifier (p=0.02). As indicated in Table 3 and confirmed in Table 4, “having a child younger than 11 years at home” had a strong association between female sex and overweight/obesity. All the analyses were repeated controlling for both physical activity and the upper tertile of the neuroticism index (NEO-PI-R). The results indicate the same significant interactions (data not shown).

## DISCUSSION

We tested the possible behavioural, biological and sociodemographic determinants of BMI in men and women separately and then compared the results to understand the weight differences between the study men and women.

The results indicate that, in all the strata, female sex was strongly and significantly associated with overweight/obesity even after controlling for the available behavioural confounders. However, the results do indicate that the effect of female sex on overweight/obesity was modified, and is less strong, in homes where children were sharing meals without any contribution. In contrast, urban residence was determined to modify the effect of female sex on underweight. The females of the farms, rural areas, and the informal sector were less likely to be underweight, and the urban/upper urban females were more likely to be underweight.

Except for sex, no other variables investigated in the study were associated with an increased risk for overweight/obesity. This implies that overweight/obesity may rather be due to an inherent susceptibility than to an external factor. The most prominent theories regarding obesity susceptibility state that the increased lipid storage capabilities may have been positively adaptive in populations undergoing numerous periods of famine, thus resulting in selection for the ‘thrifty genotype’ (39-41). The apparent increase in obesity/overweight observed in such populations is, therefore, merely the outcome of the interaction of this ancestral genotype with a food-rich environment (42) and insulin resistance (43).

The ‘thrifty gene’ hypothesis is one of the major hypotheses given for the global epidemic of obesity but it has come under fire in recent years due to lack of strong candidates for the ‘thrifty gene’ (44), and the increased evidence that the selective forces central to this hypothesis may not have been as strong as previously hypothesized (45). Furthermore, this hypothesis has been mainly applied to the European-descended populations, and its application to understanding the causes of increased obesity in the African populations may, thus, not be appropriate. This is highlighted in the current analysis due to the fact that the overweight/obese phenotype is mainly observed in females whereas the males present with normal to underweight phenotypes, although they have been exposed to similar selective forces, i.e. periods of famine.

**Table 2.** Baseline characteristics for men and women: prevalence (%)

Variable	Men (n=530)	Women (n=795)
Current smoker*	65.5	20.1
Level of education		
None**	28.6	21.5
≤10^th^ grade**	32.8	39.6
10^th^ grade and above	38.5	38.9
HIV infection status	13.0	10.9
Age-group (years)		
25-<35	34.5	35.1
35-<45	24.9	29.4
45-<55	24.3	22.5
55-<65	16.2	13.0
Employed*	60.7	42.1
Intake of alcohol above the recommended levels*,‡	22.8	6.9
Child aged <11 years at home*	50.4	63.4
Urbanization level		
Rural (under tribal law)	25.7	30.4
Commercial farm areas	16.8	15.7
Informal settlements (squatter camps)	16.6	16.6
Urban (established housing in townships)	31.9	28.1
Upper urban (main town areas)	9.1	9.2

*p<0.001;

*p<0.05;

‡Based on gender-specific guidelines: men >30 g/day and women >15 g/day (31)

Observations from the results of this study lead to the elucidation of the possible causes of sex differences in overweight/obesity by investigating sex-specific molecular pathways. The first evidence for the sex-specific molecular pathways being responsible for the effects observed in this study can be gleaned from the fact that the overweight/obesity risk in the female black South Africans was modified in households with a child aged less than 11 years. Besides, the fact that this was used as an indicator of sharing a meal with children in this study, it might also be an indicator of a process where major hormonal changes occur in the female participants as the majority were still in their reproductive age. Thus, it is plausible to assume that the changes in estrogen homeostasis that occur during this period will affect all the processes controlled by hormones, such as leptin. Leptin is an adipocyte-secreted hormone (46), which acts as an indicator of the level of fat storage (47) and regulates satiety, appetite, and weight in humans via activation of the specific hypothalamic pathways (48,49). The baseline expression of this hormone is higher in women than in men, and it has been determined that treatment of adipocytes with estradiol resulted in an increased leptin expression in women whereas a similar effect was not observed in men (50). Similarly, it was determined that the testosterone levels correlate inversely with the leptin levels in both elderly and young men (51). Thus, the major sex hormones, such as estrogens and testosterone, seem to be central to the regulation of leptin and, in turn, weight management.

Another possibility might be the association between the short *allele* of the serotonin transporter gene, which was determined to be an independent risk factor for obesity in a population of European ancestry (52), and high levels of anxiety. Neuroticism is a personality trait particularly associated with depressive and anxiety disorders. Results of a literature review by Verhagen indicate a strong relationship between gender and neuroticism and also that females scored higher than males on neuroticism indices (53). These gender differences could suggest differences in heritability between the sexes. However, research regarding gender differences in the action of the serotonin transporter gene is still inconsistent. From a behavioural genetics perspective, the findings may reflect a unique interaction among genes, gender, and the environment (54). Epigenetics propose that the environmental factors may lead to changes in expression of genes.

The black South African population belongs to the macrohaplogroup L, which is the ancestral population from which all human populations originated (55). Thus, this population is not only one of the most ancient but it also harbours the highest levels of genetic variation. Upon further investigation, it may, therefore, arise that the explanation for the disparity between the sexes in terms of BMI distribution in the black South African population may be a more complex and nuanced interplay of factors.

**Table 3.** Stratum-specific odds ratios comparing females to males adjusting for smoking

Variable	Overweight/obesity		Underweight	
	OR	95% CI	OR	95% CI
Full sample	5.07	3.77-6.8	0.67	0.44-1.02
No education	5.42	2.90-10.15	0.63	0.31-1.29
Some education	5.15	3.65-7.26	0.69	0.41-1.17
Job at the moment	5.04	3.38-7.50	0.57	0.27-1.20
No job at the moment	6.84	4.14-11.30	0.64	0.37-1.09
Urban and upper urban	5.87	3.68-9.36	1.97	0.92-4.22
Farm, rural area, and informal sector	6.10	4.00-9.31	0.44	0.26-0.75
Child aged <11 years at home	3.67	2.51-5.36	0.57	0.31-1.05
No child aged <11 years at home	7.80	4.79-12.70	0.86	0.48-1.56
Age-group (years)				
25-<35	6.34	3.48-11.53	0.66	0.32-1.38
35-<45	5.94	3.33-10.61	0.46	0.17-1.22
45-<55	3.28	1.90-5.68	0.79	0.36-1.72
55-<65	7.26	3.27-6.12	0.72	0.26-2.00

CI=Confidence interval;

OR=Odds ratios

**Table 4.** Logistic models testing for interaction terms

Modelling the association with overweight/obesity					Modelling the association with underweight			
Testing for interaction between female gender and having a child aged <11 years at home								
	β	p value	OR	95% CI	β	p value	OR	95% CI
Intercept	-1.27	<0.01			-0.44	0.33		
Female gender (=Var 1)	0.62	0.16	1.86	0.79, 4.40	-0.91	0.16	0.40	0.11, 1.43
Current smoker (=Var 2)	0.70	<0.01	2.02	1.51, 2.70	-0.79	<0.01	0.45	0.30, 0.69
Child (<11 years) in the household (=Var 3)	-0.73	<0.01	0.48	0.29, 0.78	0.21	0.35	1.23	0.79, 1.92
Interaction Var 1 * Var 3	0.70	0.02	2.01	1.12, 3.62	0.37	0.36	1.45	0.66, 3.18
Testing for interaction between female gender being aged 45-<55 years								
	β	p value	OR	95% CI	β	p value	OR	95% CI
Intercept	-1.10	0.51			0.03	0.75		
Female gender (=Var 1)	0.71	0.57	2.04	0.67, 6.25	-0.06	0.14	0.40	0.11, 1.43
Current smoker (=Var 2)	0.72	0.15	2.06	1.53, 2.77	-0.77	<0.01	0.45	0.30, 0.69
Age 45-<55 years (=Var 3)	-0.73	0.26	0.48	0.29, 0.81	-0.10	0.72	1.23	0.79, 1.92
Interaction Var 1 * Var 3	0.54	0.32	1.71	0.91, 3.22	-0.20	0.66	1.45	0.66, 3.18
Testing for interaction between female gender and urban residence								
	β	p value	OR	95% CI	β	p value	OR	95% CI
Intercept	-0.89	0.05			-0.15	0.75		
Female gender (=Var 1)	1.56	<0.01	4.75	1.84, 12.28	1.05	0.15	2.85	0.69, 11.69
Current smoker (=Var 2)	0.61	<0.01	1.83	1.36, 2.47	-0.85	<0.01	0.43	0.28, 0.65
Urban residence (=Var 3)	-0.86	<0.01	0.42	0.26, 0.69	0.07	0.78	1.07	0.67, 1.69
Interaction Var 1 * Var 3	0.14	0.65	1.15	0.64, 2.06	-0.85	.042	0.43	0.19, 0.97

OR=Odds ratio;

CI=Confidence interval;

Var=Variable;

*Association between Variable 1 and Variable 3

### Conclusions

The findings of the present study highlight the importance of understanding the molecular roots of the sex/gender-specific variability in BMI distribution in black South African adults. This process is central to the future development of treatment and prevention programmes against overweight/obesity in this population.

## ACKNOWLEDGEMENTS

The authors gratefully acknowledge the THUSA research team and the leader Prof. H.H. Vorster for making the data available.
